# Selective reduction of the light peak-to-dark trough ratio in reticular macular disease: an electrooculography study

**DOI:** 10.3389/fnins.2026.1865041

**Published:** 2026-07-14

**Authors:** Lin Liu, Lu Cheng, Qi Ren, Zhe Chu, Hao Cheng

**Affiliations:** 1 Department of Ophthalmology, The First Affiliated Hospital of Guangzhou Medical University, Guangzhou, China; 2 Ophthalmic Center, The Second Affiliated Hospital of Guangzhou Medical University, Guangzhou, Guangdong, China; 3 Department of Ophthalmology, The First Affiliated Hospital, Sun Yat-sen University, Guangzhou, China; 4 Shandong Provincial Key Laboratory of Ophthalmology, Eye Institute of Shandong First Medical University, Qingdao, China

**Keywords:** age-related macular degeneration (AMD), electrooculography (EOG), reticular macular disease, reticular pseudodrusen, retinal pigment epithelium (RPE), RPE-photoreceptor complex, subretinal drusenoid deposits

## Abstract

**Purpose:**

To compare electrooculogram (EOG) parameters between eyes with reticular macular disease (RMD) and eyes with early-to-intermediate age-related macular degeneration (AMD) without reticular pseudodrusen (RPD), and to assess whether RMD is associated with generalized RPE–photoreceptor complex dysfunction.

**Methods:**

In this observational cross-sectional study, 42 eyes from 21 patients were analyzed (without-RPD group: 28 eyes; RMD group: 14 eyes). RMD was diagnosed using multimodal imaging (infrared reflectance and SD-OCT). EOGs were recorded with the RETI-port/scan 21 system according to ISCEV standards. Extracted parameters included dark trough (DT), light peak (LP), light rise time (Light Rise), and the LP:DT ratio. Group comparisons used the Wilcoxon rank-sum test. Linear regression evaluated the association between RMD and LP:DT ratio with and without adjustment for age.

**Results:**

Age did not differ between groups (median 69 vs. 72 years; *P* = 0.5). Absolute standing potentials were similar (DT: 598 vs. 681 μV, *P* = 0.2; LP: 1,092 vs. 1,150 μV, *P* = 0.8). Light rise time was also similar (8.00 [IQR 7.50–9.00] vs. 8.00 [IQR 7.00–9.00] min; *P* = 0.6). In contrast, the LP:DT ratio was significantly reduced in RMD (1.65 [1.50–1.80]) compared with without-RPD controls (1.90 [1.80–2.10]; *P* = 0.0043). RMD was independently associated with a lower LP:DT ratio (β = −0.25, 95% CI [−0.41 to −0.09]; *P* = 0.004).

**Conclusion:**

Compared with AMD eyes without RPD, RMD eyes demonstrate a selectively reduced LP:DT ratio without a change in absolute standing potentials or light rise timing. This EOG pattern supports attenuated light response consistent with generalized dysfunction of the RPE–photoreceptor complex in RMD.

## 1 Introduction

Age-related macular degeneration (AMD) is a degenerative retinal disease characterized by disruptions in the outer retinal and choroidal microenvironment. In its advanced stages, AMD leads to central vision loss through atrophic or neovascular complications and stands as one of the leading causes of severe, irreversible visual impairment worldwide. The global prevalence of AMD is approximately 8.69% among individuals aged 45–85 years, with the patient population projected to reach 288 million by 2040 (Chinese Vitreo-Retina Society of Chinese Medical Association, and Fundus Disease Group of Chinese Ophthalmologist Association., 2023). Reticular macular disease (RMD) is increasingly recognized as a distinct AMD phenotype, characterized by reticular pseudodrusen (RPD) accompanied by perfusion dysfunction in the choriocapillaris (Chu et al., 2023a,b; Diao and Cheng, 2021; Li et al., 2023; Smith et al., 2009; Thomson et al., 2022; Wei et al., 2023; Ye and Cheng, 2019). While both RPD and typical drusen are considered precursors to advanced AMD, they differ significantly in appearance, histology, and prognosis (Curcio et al., 2013; Rabiolo et al., 2017; Thomson et al., 2022; Wu et al., 2022). On infrared retinal imaging, RPD manifests as a hyporeflective network; lesions breaching the external limiting membrane appear as hyperreflective targets within hyporeflective spots. On optical coherence tomography (OCT), RPD appears as subretinal drusenoid deposits (SDD) (Zweifel et al., 2010). Histologically, RPDs are extracellular deposits located in the subretinal space between the neurosensory retina and the retinal pigment epithelium (RPE). They form piles or clumps adjacent to photoreceptor outer segments, either isolated or fused atop the RPE. When fused, adjacent lesions interconnect to form a reticular pattern (Zweifel et al., 2010). RPD presence is often associated with shortening of photoreceptor outer segments. Notably, while rod outer segments may become embedded within the deposits, cone outer segments typically do not. The RPE cells exhibit abnormal size and morphology and may migrate around, but not into, the RPD (Greferath et al., 2016). Biochemically, RPD shares several components with drusen, such as apoE, complement factor H, and vitronectin, but differs in lipid composition; RPD contains lower levels of apoB and apoA-1, consists solely of free cholesterol without cholesteryl esters, whereas drusen contain both. The precise pathogenesis of RPD remains unclear, though it is hypothesized that age-related loss of polarity in RPE transport mechanisms – including mislocalization of Na^+^/K^+^-ATPase and disruption of vectorial secretion – leads to the apical excretion and subsequent subretinal deposition of metabolites. This polarity loss may cause metabolites normally excreted toward the choroid to be redirected into the subretinal space, accounting for the characteristic localization of SDD above the RPE. Regardless of the mechanism, the presence of RPD clearly disrupts the subretinal microenvironment and is associated with morphological abnormalities in adjacent RPE and rod cells, potentially interfering with visual signal transduction. Recent studies indicate that mesopic visual sensitivity is generally reduced in eyes with large drusen and concomitant RPD compared to eyes without RPD, with deficits correlating with the extent of RPD (Kumar et al., 2022). Therefore, characterizing the outer-retinal electrophysiological profile, including the integrated light response that reflects RPE–photoreceptor interaction, in RMD-affected eyes is of critical importance.

The electrooculogram (EOG) assesses the integrity of the RPE and photoreceptors by measuring the corneoretinal standing potential during dark adaptation (DA) and light adaptation (LA). This standing potential, positive at the cornea, is an indirect measure of the transepithelial potential (TEP)–the voltage difference across the electrochemically insulated tight junctions between the apical and basolateral membranes of the RPE (Creel, 2019; Djukanovic et al., 2024). Although the EOG dark trough (DT) originates primarily from the RPE, the subsequent light rise requires the functional integrity of both the RPE and the neurosensory retina. Under bright light, bestrophin channels in the endoplasmic reticulum interact with L-type calcium channels on the basolateral membrane to regulate calcium release. This increase in intracellular free calcium triggers the opening of calcium-dependent chloride channels on the basolateral membrane, increasing chloride conductivity and leading to membrane depolarization and an elevation in TEP. Given the inter-individual variability in absolute voltage (Thavikulwat et al., 2015), the clinical evaluation of EOG relies mainly on the light rise and the Arden ratio (LP:DT ratio)–the ratio of the light peak (LP) amplitude to the DT amplitude (Arden and Constable, 2006). A normal light rise exceeds 170%, necessitating functional photoreceptor contact with a healthy RPE. An LP:DT ratio of <1.7 is considered abnormal (Creel, 2019).

Electrooculogram is sensitive to rod dysfunction, with the light rise typically severely reduced in widespread photoreceptor degenerations such as retinitis pigmentosa. This rod sensitivity is particularly relevant to RMD given that SDDs preferentially localize to rod-rich perifoveal and peripapillary regions, whereas drusen localize to cone-rich areas (Curcio et al., 2013). The combination of rod-predominant SDD distribution and the EOG’s sensitivity to rod-driven signals raises the possibility that RMD may produce discernible EOG changes even when full-field photoreceptor degenerations are absent.

Given associations between RMD, RPE morphological abnormalities, and rod-rich SDD distribution, we hypothesized that RMD eyes would demonstrate characteristic EOG abnormalities compared with early-to-intermediate AMD eyes without RPD. To test this hypothesis, we compared the electrophysiological profiles of eyes with RMD and those with early-to-intermediate AMD without RPD to characterize outer-retinal electrophysiological function as reflected by EOG in reticular macular disease.

## 2 Materials and methods

This observational cross-sectional study was conducted at the First Affiliated Hospital of Guangzhou Medical University. The study followed the principles outlined in the Declaration of Helsinki and was approved by the Ethics Committee of the First Affiliated Hospital of Guangzhou Medical University (Approval No. ES-2024-157-01). A total of 42 eyes from 21 patients were analyzed.

### 2.1 Eligibility criteria

**FIGURE 1:**
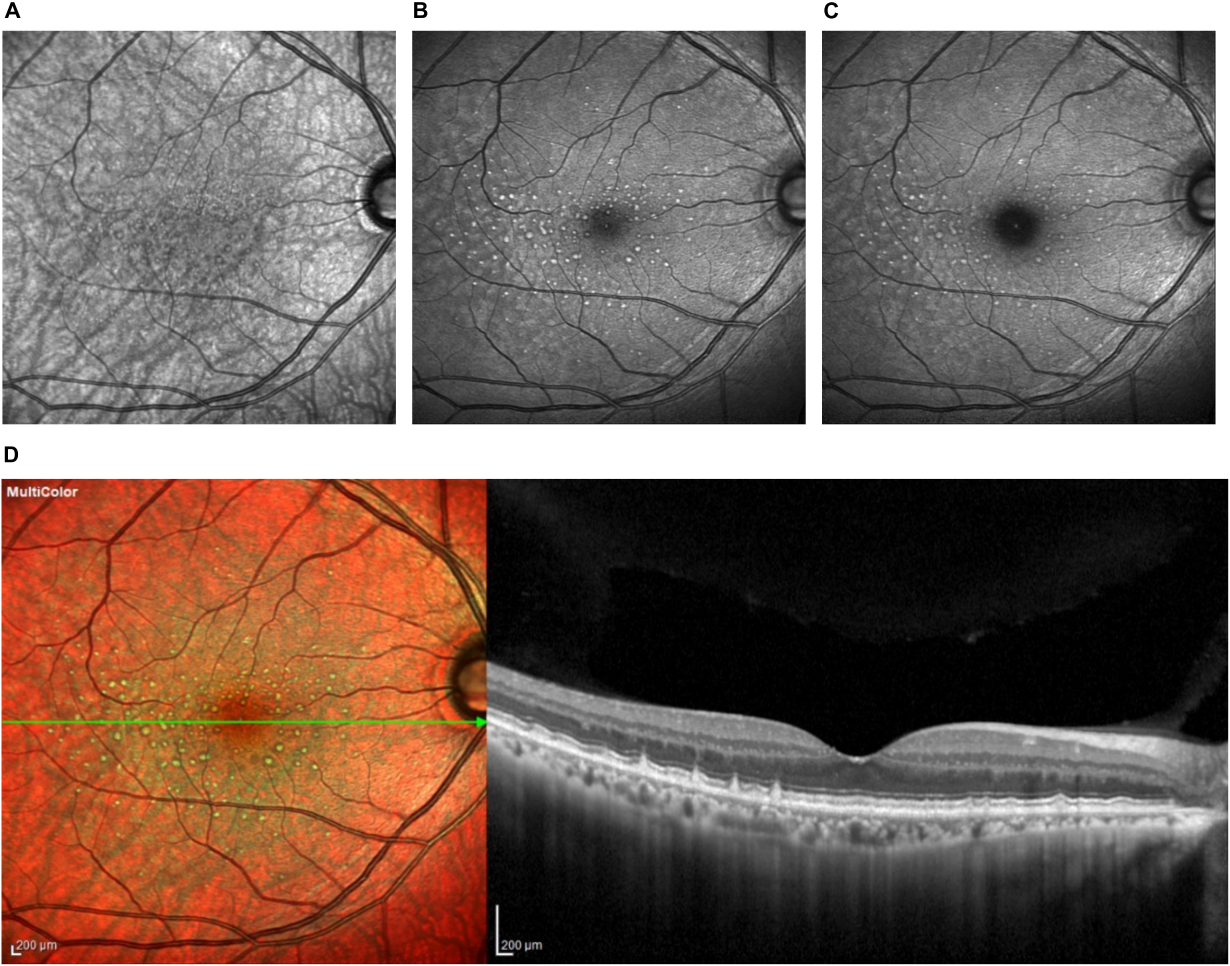
Reticular macular disease (RMD) on multispectral, confocal scanning laser ophthalmoscopy. This imaging modality contains three color-selective laser image information: **(A)** near infrared reflectance at a wavelength of λ = 820 nm, **(B)** green reflectance at a wavelength of λ = 515 nm, and **(C)** blue reflectance at a wavelength of λ = 488 nm. **(D)** The information of the three reflection images were merged into one multispectral image. RPD with a low-reflection network distribution is shown in each modality, and the part that broke through the outer membrane is shown as a high reflection point at the center of the low-reflection spot. RPD appeared as SDD on OCT section. OCT, optical coherence tomography; RMD, reticular macular disease; RPD, reticular pseudodrusen; SDD, subretinal drusenoid deposits.

Age-related macular degeneration was diagnosed using criteria developed by The Age-Related Eye Disease Study Research Group (AREDS) in 2013 (Ferris et al., 2013; Flaxel et al., 2020). The diagnosis of RMD is as follows: (1) Infrared imaging of the fundus revealed reticular low-reflection spots on the background with relatively high reflection. (2) Spectral domain-OCT images showed the presence of clusters of granular hyperreflective deposits located above the RPE, which compressed the ellipsoid reflection zone with wavy uplift or interruption of continuity or breached the outer membrane boundary to the outer nuclear layer (Figure 1; Guymer et al., 2020; Pumariega et al., 2011; Smith et al., 2009; Zweifel et al., 2010).

Inclusion criteria. (1) Patients diagnosed with RMD will be included in the RMD group; at least one eye met both of the above criteria, and the reticular lesions covered more than 1/2 of the retina above the macular area or more than 1/3 of the retinal area and were dispersed in the papillary temporal and nasal retinas (Yun et al., 2016). (2) Patients with early-to-intermediate AMD (without RPD) will be included in the non-RMD group, including a combination of multiple small drusen, few intermediate drusen (63–124 μm in diameter), or mild RPE abnormalities (AREDS category 2); numerous intermediate drusen and/or at least one large druse (≥125 μm in diameter) (part criteria of AREDS category 3) (Flaxel et al., 2020). (3) Considering the diagnostic criteria for AMD, subjects over the age of 50 years will be included (Flaxel et al., 2020). All eyes in this group had clinically confirmed AMD; no healthy controls were enrolled.

Exclusion criteria. (1) Advanced-stage AMD will be excluded, considering that advanced AMD will be one of the outcome events in this study. (2) Stargardt disease, Best disease, and other known RPE–photoreceptor injury diseases. (3) Refractive media opacification, such as severe vitreous opacity and cataracts, may affect retinal light perception during EOG examination.

### 2.2 Electrooculography recording

**FIGURE 2:**
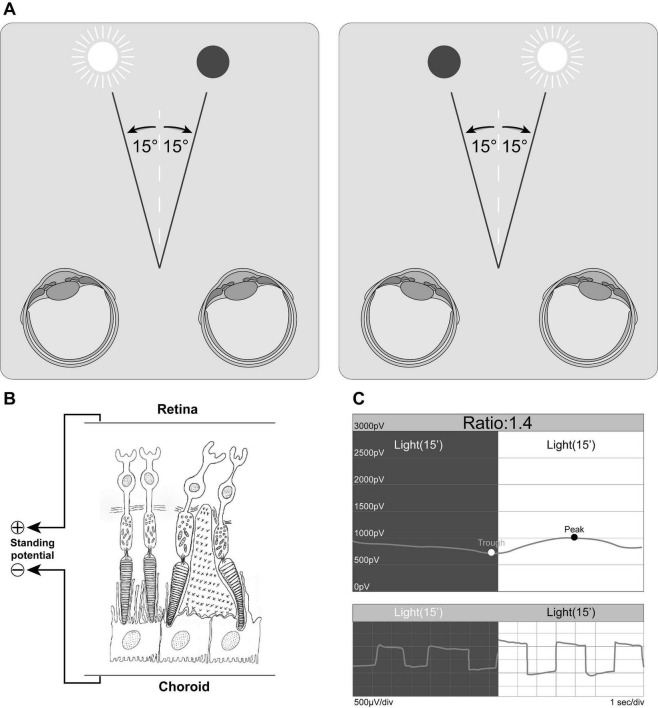
Electrooculogram (EOG) detection. **(A)** The participants fixed their heads and moved their eyes to track a fixed light on their left or right side. Recording of oscillating potentials. **(B)** Potential generation of the EOG. RPE cells generate voltage from vertex to base (transepithelial potential, TEP) due to different ionic permeability properties on membrane surfaces in different directions and the presence of impermeable tight connections between the basolateral membrane and the apical membrane. A change in impedance between the apical and basolateral membranes, or a change in membrane potential, can affect the amplitude of TEP, thereby affecting the potential difference between the anterior segment and the posterior segment of the eye. **(C)** This change can be clinically recorded by EOG.

The EOG was determined by the visual electrophysiology instrument RETI-Port/Scan 21 (Roland Consult Stasche & Finger GmbH, Brandenburg) for each patient’s eyes. The detection methods were based on the ISCEV Visual Electrodiagnostic Procedures 2017 edition (Constable et al., 2017a; Robson et al., 2018; Figure 2). Prior to testing, pupils were dilated to a diameter of at least 6 mm using 0.5% tropicamide and 0.5% phenylephrine hydrochloride. The skin was cleaned with abrasive paste to ensure low impedance (<5 kΩ), and Ag/AgCl skin electrodes were placed at the outer and inner canthi of each eye, with a ground electrode attached to the forehead (Fpz). The recording protocol consisted of a 15-min dark adaptation phase followed by a 15-min light adaptation phase. During the light phase, a standardized full-field background light (Ganzfeld bowl) with a luminance of 100 cd/m^2^ was used. Patients were instructed to perform horizontal saccadic eye movements over an angle of 30° guided by alternating fixation lights, with a frequency of one saccade per second (1 Hz).

### 2.3 Data processing and statistical analysis

The standing potential was continuously monitored throughout the recording. The following parameters were extracted for analysis: (1) DT: the minimum voltage amplitude reached during the dark phase; (2) LP: the maximum voltage amplitude reached during the light phase; (3) Light Rise: the time interval from the DT to the LP; and (4) LP:DT ratio: the ratio of the LP amplitude to the DT amplitude.

Statistical analysis was performed using R software (version 4.5.0). Continuous variables were presented as median [interquartile range, IQR] due to the non-normal distribution of electrophysiological data. Comparisons between the RMD group and the without-RPD group were performed using the Mann-Whitney U test (Wilcoxon rank-sum test). *P* < 0.05 was considered statistically significant.

## 3 Results

### 3.1 Demographic and baseline electrophysiological characteristics

A total of 42 eyes were included in the analysis, comprising 28 eyes in the without-RPD group and 14 eyes in the RMD group. The demographic and baseline electrophysiological characteristics are summarized in Table 1. There was no significant difference in age between the two groups (median: 69 vs. 72 years, *P* = 0.5). Similarly, the absolute amplitudes of the standing potential at the DT and LP phases did not differ significantly between groups (*P* = 0.2 and *P* = 0.8, respectively).

**TABLE 1:** Characteristics of the study sample.

Characteristic	Without-RPD *N* = 28^1^	RMD *N* = 14^1^	*P*-value^2^
Age (years)	69 (65, 73)	72 (58, 76)	0.5
Dark trough (μV)	598 (490, 686)	681 (491, 847)	0.2
Dark time (min)	7.00 (6.50, 7.50)	7.50 (6.00, 8.50)	0.7
Light peak (μV)	1,092 (943, 1,346)	1,150 (928, 1,400)	0.8
Light rise (min)	8.00 (7.50, 9.00)	8.00 (7.00, 9.00)	0.6

^1^Median (Q1, Q3).

^2^Wilcoxon rank sum test; Wilcoxon rank sum exact test.

### 3.2 Similarity in light rise time

**FIGURE 3:**
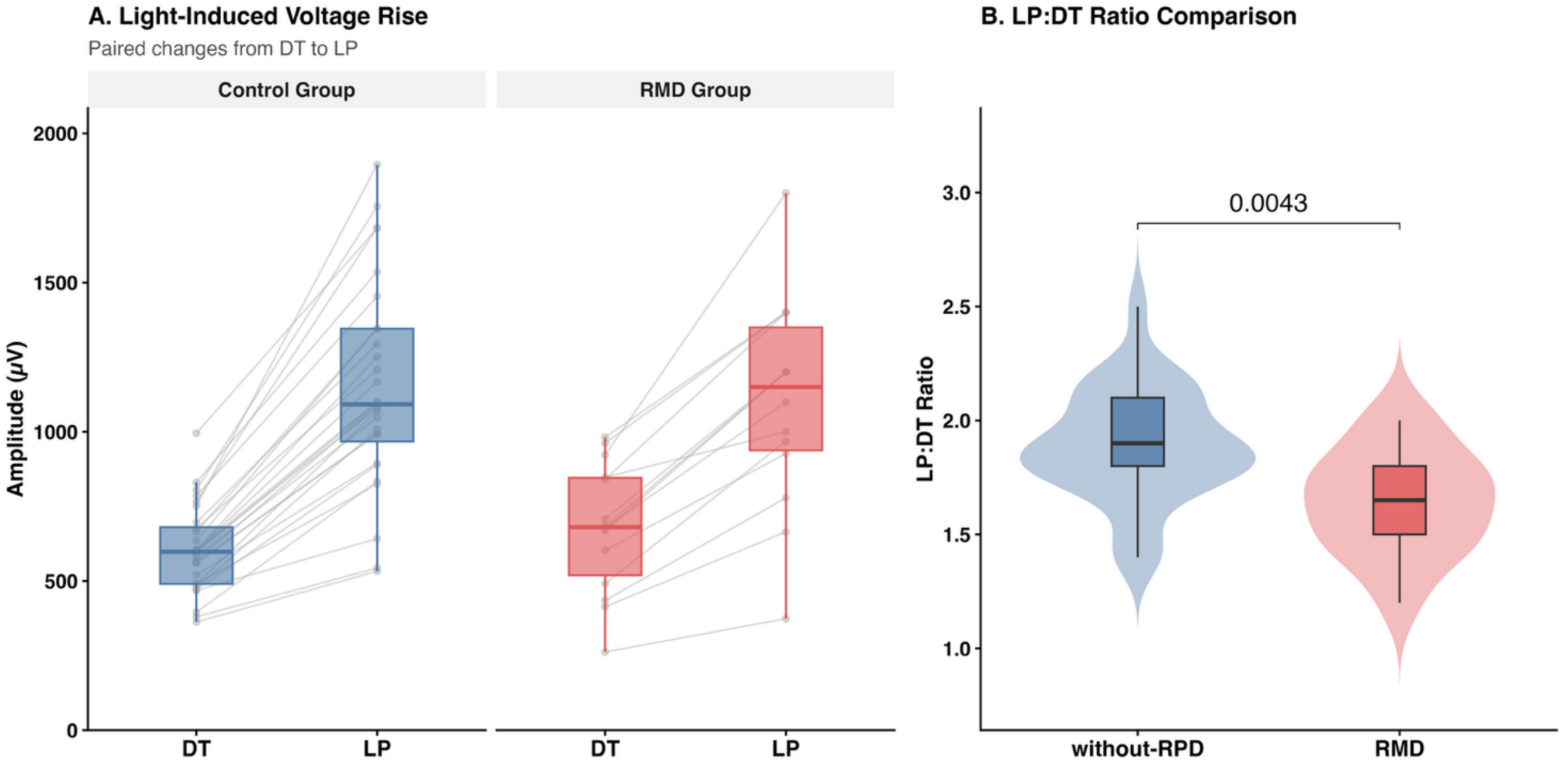
Electrophysiological characteristics of reticular macular disease (RMD). **(A)** Light-induced voltage rise. Paired boxplots illustrate the dynamic change in standing potential from the dark trough (DT) to the light peak (LP) for each eye. Individual gray lines connect paired measurements, visualizing the physiological voltage elevation in both the without-RPD (blue) and RMD (red) groups. **(B)** LP:DT ratio comparison. Violin plots displaying the distribution density of the LP:DT ratio. The RMD group exhibited a significantly reduced ratio compared to the without-RPD group (*P* = 0.0043, Wilcoxon rank sum test). The internal boxplots indicate the median and interquartile range.

Consistent with the findings in absolute voltages, the temporal dynamics of the light response were also similar between the groups. The Light Rise showed no significant difference between the without-RPD and RMD groups (median: 8.00 [IQR: 7.50–9.00] vs. 8.00 [IQR: 7.00–9.00] min, *P* = 0.6) (Figure 3A and Table 1). This indicates that light-rise timing was preserved in RMD eyes with attenuation confined to response magnitude.

### 3.3 Impaired LP:DT ratio

Despite the similarity in absolute baseline voltages and peak timing, the functional relationship between the light and dark potentials was significantly altered. The median LP:DT ratio was significantly reduced in the RMD group (1.65 [IQR: 1.50–1.80]) compared to the without-RPD group (1.90 [IQR: 1.80–2.10]; *P* = 0.0043, Wilcoxon rank sum test) (Figure 3B). This suggests that the proportional rise in potential relative to the baseline is blunted in RMD.

### 3.4 Independent association of RMD with EOG dysfunction

**FIGURE 4:**
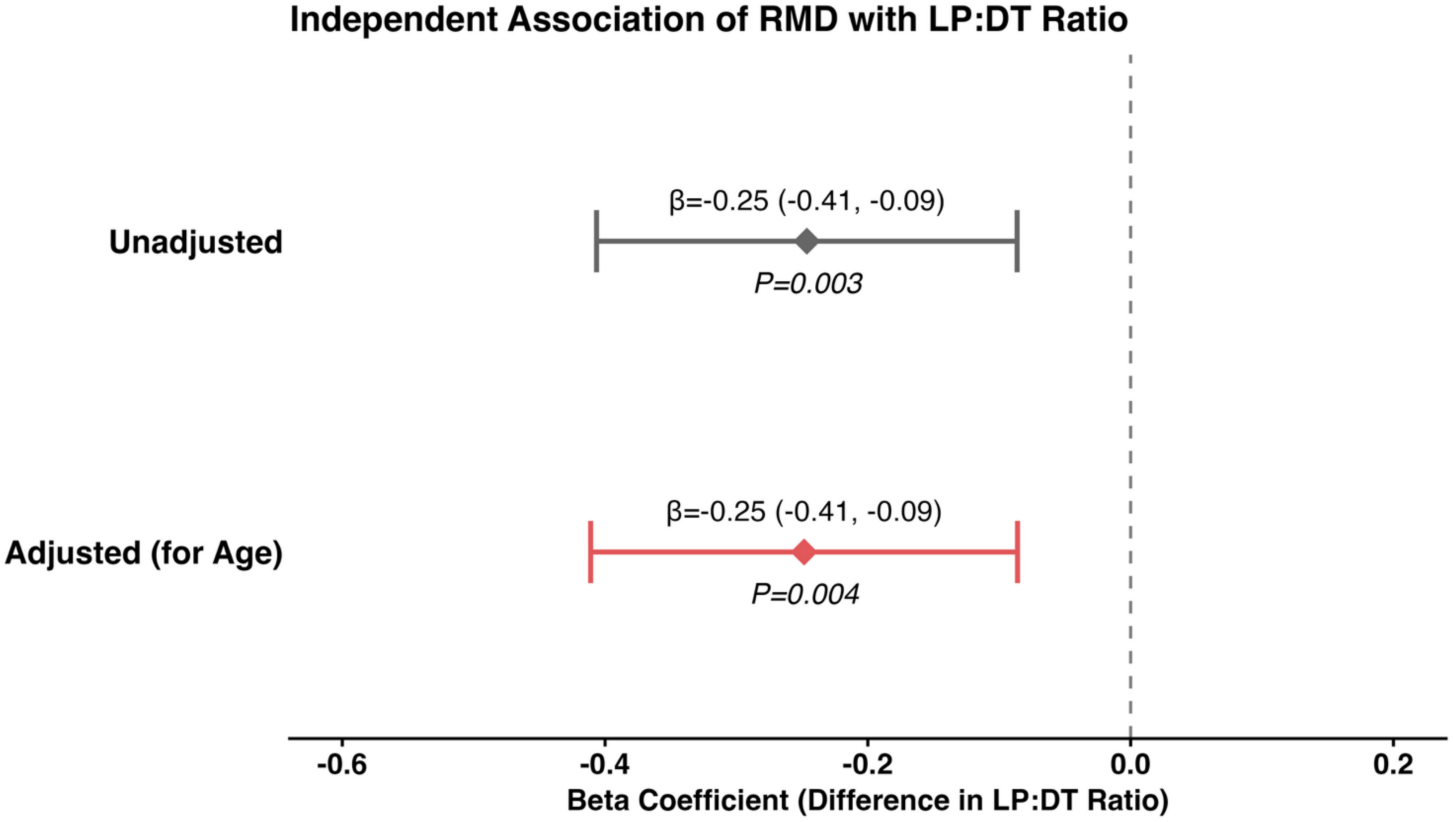
Independent association of RMD with LP:DT ratio. Forest plot displaying the results of linear regression analyses quantifying the effect of the RMD phenotype on the LP:DT ratio. The *x*-axis represents the Beta coefficient (difference compared to the without-RPD group), with error bars indicating 95% confidence intervals.

To determine whether the observed reduction in the LP:DT ratio was independently associated with the RMD phenotype or confounded by age, we performed a linear regression analysis (Figure 4). In the unadjusted model, the presence of RMD was associated with a significant decrease in the LP:DT ratio (β = −0.25, 95% CI: −0.41 to −0.09; *P* = 0.003). After adjusting for age, this negative association remained stable and statistically significant (β = −0.25, 95% CI: −0.41 to −0.09; *P* = 0.004). These results indicate that the EOG dysfunction, characterized by a lower LP:DT ratio, is an intrinsic feature of RMD independent of aging.

## 4 Discussion

In this study, we compared EOG findings in 14 eyes with RMD and 28 eyes with early-to-intermediate AMD without RPD. Three findings together define a characteristic EOG profile in RMD. First, absolute standing potentials at the dark trough (DT) and light peak (LP) did not differ significantly between groups, indicating that overall standing potential amplitude was largely preserved. Second, light rise timing was comparable between groups (median, 8.0 min in both), consistent with a preserved time course of the light rise rather than a prolonged time-to-peak. Third, despite similar absolute amplitudes and preserved timing, the LP:DT ratio was significantly reduced in RMD and remained independently associated with the RMD phenotype after adjustment for age. Together, these findings indicate a blunted EOG light rise–a disproportionately reduced light-evoked increase relative to the dark trough–rather than a global reduction in standing potential or delayed light rise timing.

The LP:DT ratio is a standardized summary measure of the EOG light response that normalizes the light peak to the preceding dark trough. The ISCEV standard defines a normal range of 1.7–4.3 (Constable et al., 2017a), and a meta-analysis reported a mean value of approximately 2.35 (Constable et al., 2017b). In our cohort, the median LP:DT ratio was 1.65 (IQR 1.50–1.80) in the RMD group versus 1.90 (IQR 1.80–2.10) in the without-RPD group (*P* = 0.0043). Importantly, the lack of group differences in absolute DT and LP voltages suggests that the primary abnormality in RMD is not a uniform reduction in standing potential, but rather a disproportionately smaller light-evoked increase relative to the dark baseline. Because ratio metrics reduce inter-individual variability in absolute voltage recordings, the LP:DT ratio may capture outer-retinal dysfunction that is not apparent when DT or LP amplitudes are considered alone. The EOG light rise reflects the integrated interaction between photoreceptor-driven signaling and RPE electrophysiology. The combination of a normal time-to-peak with an attenuated LP:DT ratio indicates that the light response in RMD is not delayed but rather reduced in magnitude. This pattern points to a generalized reduction in RPE–photoreceptor complex functional responsiveness rather than a slowed response process.

Comparisons with typical AMD also help contextualize our findings. Prior reports suggest that EOG parameters in early-to-intermediate AMD may remain relatively stable and become consistently abnormal mainly in the presence of geographic atrophy (Walter et al., 1999). The observation that RMD eyes demonstrate a significantly reduced LP:DT ratio compared with AMD eyes without RPD–despite similar age distribution, similar absolute standing potentials, and preserved timing–supports the notion that RMD represents a distinct AMD phenotype with more diffuse functional compromise of the RPE–photoreceptor complex. The earlier and more pronounced functional deficit in RMD relative to non-RPD AMD may reflect the distribution and composition of the deposits: SDDs are widespread across the posterior pole and preferentially located in rod-rich regions to which the EOG light rise is particularly sensitive (Curcio et al., 2013), whereas drusen are more focal and concentrated in the cone-rich macula. Furthermore, the high free-cholesterol content of SDDs may be more disruptive to RPE ion channel function than the esterified cholesterol found in drusen (Greferath et al., 2016; Zweifel et al., 2010).

Complementing EOG, prior electrophysiological studies using ERG have reported functional deficits in RMD/RPD. The electroretinogram (ERG) records the mass electrical response of the retina, primarily reflecting light-induced currents from longitudinally arranged neurons such as photoreceptors and bipolar cells. Previous studies on the ERG characteristics of RMD patients have reported varying degrees of reduction in ERG parameters (Alten et al., 2014; Diao et al., 2019; Kong et al., 2018; Savastano et al., 2022). Flash ERG (fERG) responses were attenuated in eyes with diffuse RPD compared to healthy eyes (Kong et al., 2018), and fERG amplitude correlates with ellipsoid zone disruption and outer retinal thinning with greater reduction in RPD eyes than in those with drusen (Savastano et al., 2022). Multifocal ERG studies further demonstrated localized amplitude reduction in RPD-affected areas with progressive decline (Alten et al., 2014), and regional analyses identified functional loss in specific macular sectors in RMD (Diao et al., 2019). Viewed together with our EOG findings, these data support a broad functional impairment involving both the RPE-related standing potential response and retinal neural function in the RMD/RPD phenotype.

This study has limitations. The sample size is modest, and the analysis was cross-sectional, precluding assessment of whether the observed EOG changes predict longitudinal structural progression. Potential confounders affecting retinal illumination (e.g., media opacity) cannot be completely excluded, although ratio-based metrics reduce some inter-individual variability and the exclusion of eyes with significant cataract mitigated this concern. OCTA data were not available to explore the relationship between choriocapillaris perfusion and the blunted light rise. Future longitudinal studies incorporating OCTA are warranted to determine whether reduced LP:DT ratio predicts structural progression to geographic atrophy or serves as a functional biomarker of RPE stress in clinical trials before irreversible structural damage occurs.

In summary, the RMD EOG profile is defined by a reduced magnitude of light-evoked response without a delay in processing: light rise time and absolute standing potentials are preserved, while the LP:DT ratio is significantly and independently reduced. These findings indicate a functional dampening of the EOG light response in RMD, consistent with generalized but selective dysfunction of the RPE–photoreceptor complex.

## Data Availability

The data analyzed in this study is subject to the following licenses/restrictions: The datasets generated and/or analysed during the current study are available from the corresponding author on reasonable request. Requests to access these datasets should be directed to HC, chrischenghao@gzhmu.edu.cn.
